# Moon Insight: a conceptual framework for future lunar exploration

**DOI:** 10.1093/nsr/nwag474

**Published:** 2026-08-05

**Authors:** Yang Liu, Yongliao Zou, Chi Wang

**Affiliations:** National Space Science Center, Chinese Academy of Sciences, China; National Space Science Center, Chinese Academy of Sciences, China; National Space Science Center, Chinese Academy of Sciences, China

## Abstract

The Moon Insight conceptual framework aims to map lunar lava tubes and understand their shapes and internal structures. It will use these natural underground spaces to study the Moon's interior and volcanic history, improve our understanding of how the Moon formed and evolved, and support future lunar bases, long-term human stays, scientific research, and resource use.

Lunar science has advanced significantly since Apollo era, establishing a foundational understanding of the Moon’s geological processes and evolutionary history. In particular, China’s Lunar Exploration Program has revolutionized our knowledge through a series of successful orbital, surface, and sample-return missions. Despite these achievements, the Moon’s interior structure and long-term thermal evolution remain poorly constrained, posing a major scientific challenge to reconstructing a holistic model of its overall evolution [1]. Concurrently, the paradigm of lunar exploration is undergoing a profound transition from purely scientific reconnaissance to a dual-purpose phase focused on both ‘understanding the Moon’ and ‘utilizing lunar resources’ [2]. Within this context, the establishment of permanent lunar bases to support sustained exploration and *in situ* resource utilization has emerged as a strategic priority.

The Moon’s interior structure preserves a fundamental record of early planetary differentiation and thermal evolution, providing critical constraints on models of terrestrial bodies formation and evolution. Investigating lunar interior structure involves precisely characterizing the crust, mantle, and core to resolve mass distribution and lithospheric properties resulting from the early global differentiation as the cooling of the lunar magma ocean [3]. Studying its thermal evolution addresses the temporal progression of interior heat, examining cooling rates and the factors governed the magnitude and periodicity of lunar magmatism [4]. Mare basalts, as solidified products of partial melts from the deep lunar interior, serve as primary petrological records, with their eruptive history providing critical constraints on the Moon’s thermal state. Lunar lava tubes (Text S1), formed during the solidification of lava flows offer rare access to undisturbed basaltic stratigraphy and deep-seated materials essential for characterizing the lunar interior [5]. Furthermore, their inherent structural integrity and natural shielding properties of lava tube make them primary candidates for establishing integrated scientific stations and long-term lunar bases [1,6], thereby facilitating both fundamental scientific discovery and sustainable exploration.

Previous studies have laid a solid foundation for the exploration and potential utilization of lunar lava tubes. For example, Xiao *et al.* [7] proposed a pioneering three-step strategy for lunar lava tube exploration, providing an important roadmap for the progressive identification, characterization, and *in situ* investigation of these subsurface structures. The ESA Moon Village concept [8] further highlighted the great potential of lunar lava tubes as natural shelters for sustainable human activities, while the JAXA LunaTube mission concept [9] provided a detailed robotic exploration scenario for accessing and investigating individual lava tubes. These studies collectively demonstrated the scientific and engineering significance of lunar lava tubes and have provided important guidance for subsequent exploration concepts. Building upon these previous efforts, we propose the ‘Moon Insight’ conceptual framework that further integrates lunar lava tube exploration with the strategic development roadmap of future Chinese lunar exploration missions. Rather than focusing solely on the detection or utilization of lava tubes, Moon Insight emphasizes their dual role as both scientifically valuable targets and key infrastructure for future lunar exploration. In particular, the framework expands previous concepts by establishing an integrated pathway that connects global mapping, *in situ* exploration, and lava tube-based lunar base construction, enabling lava tube to serve as long-term scientific platforms for investigating the Moon’s interior structure and evolution.

Utilizing remote sensing and in-situ methods, the roadmap comprises three sequential phases: Phase I, Global Survey & Mapping; Phase II, Detailed *in situ* Exploration; Phase III, Construction of a Long-term Lunar Base. The scientific objectives are: (i) to map the global distribution of lava tubes and the thickness of basalts and cryptomare, and to understand the dynamics of magma migration and emplacement; (ii) to characterize the internal structures and stability of lava tubes, and to decipher the scale, styles, and temporal evolution of lunar volcanism; (iii) to uncover the fine layered structures and dynamics of the lunar interior, and to build new theoretical model of lunar formation and evolution. These efforts will directly support future base construction, long-term human habitation, and ongoing scientific research and resource utilization on the Moon.

**Phase I: Global survey and mapping of lunar lava tubes and the thickness of basalts and cryptomare.** The scientific objective of Phase I is to produce the first global inventory of lunar lava tube candidates, including constraints on their spatial distribution, dimensions, and burial depths, while also providing key geological context such as the thickness of associated basaltic units and cryptomare deposits. A global survey of lava tubes, along with the thickness of basaltic and cryptomare deposits, can be achieved through an integrated, multi-dataset approach. High-resolution optical imagery is first used to identify lava tube skylights, that is, collapse pits formed by ceiling failure of subsurface voids [10,11] (see Text S1, Fig. S1), thereby constraining their spatial distribution and defining targets for further investigation. Gravity data are then used to probe subsurface structures. Because lava tubes have significantly lower density than the surrounding rocks, they generate subtle gravity anomalies [12,13] from which their geometry including length, width, height, and depth can be inferred. The most reliable method is deep-penetrating radar with high vertical resolution. Radar sounding directly identifies subsurface structures and refine geometric models by detecting dielectric contrasts between void and surrounding rock. Its ability to resolve thin layers and penetrate deeply makes it particularly effective for accurately determining lava tube thickness and internal configuration [14]. Furthermore, radar observations can constrain the thickness and subsurface structure of basaltic flows and cryptomare deposits, while visible and near-infrared spectrometer measurements can characterize their compositional variations. Together, these observations will provide critical insights into magma migration, emplacement processes, and the volcanic evolution of the Moon.

**Phase II: Detailed *in situ* exploration of lava tubes.** The scientific objective of Phase II is to characterize the internal structure and engineering stability of 2–3 high-priority lava tubes, while obtaining in situ measurements of their exposed stratigraphic sections to provide new constraints on the Moon’s volcanic history and magmatic evolution. Detailed exploration of lunar lava tubes is a prerequisite for understanding their refined structural characteristics and determining key engineering parameters for future lunar base construction [15,16]. Skylights of collapsed lava tube provides a window to investigate the interior details of lava tube. Primary engineering challenges focus on long-term structural stability, including post-formation degradation, internal temperature and radiation fluctuations, collapse mechanisms, and factors influencing overall structural integrity. These engineering concerns drive the need for *in-situ* three-dimensional reconnaissance by robots. Data gathered will resolve engineering uncertainties and ensure the feasibility of utilizing lava tubes as safe habitats. Detailed exploration also provides key support for studying lunar magmatism and thermal evolution through the fine characterization of their geological features [4,5]. *In-situ* robotic investigation will gather critical data on stratigraphic sections, mineral properties, and composition of the inner wall of a lunar lava tube cave. These integrated datasets will address important scientific questions, such as the multi-stage characteristics of lava flow activities, compositional variations in magmatic activity across different periods, and the specific emplacement mechanisms and controlling factors of lunar magma. A comprehensive analysis of these aspects, along with the global mapping of the thickness of basalts and cryptomare conducted in Phase I, will ultimately enhance our understanding of lunar magmatism and provide critical insights into the thermal evolution of the Moon. Additionally, detailed characterization of ancient regolith stratigraphic structures and laboratory analysis of returned samples can provide essential evidence for understanding lunar surface processes and space environment [17,18]. Such data are critical for addressing scientific questions concerning ancient regolith evolution and its response to space weathering. Such exploration is expected to reveal early lunar surface impact flux and preserved records of the paleo-Earth’s atmosphere and ancient solar activity captured within these lunar archives.

**Phase III: Construction of long-term lunar base.** The scientific objective of Phase III is to establish a long-term lunar base near an optimal lava tube site and deploy a multi-physics geophysical observation network to investigate the Moon’s deep interior, providing critical constraints on crustal thickness, mantle structure, and core properties. A lunar base would enable sustained, long-term scientific exploration of the Moon’s geology, resources, and environment. It would also serve as a habitat for astronauts, supporting extended missions and reducing reliance on Earth-based resupply. A lunar base could be strategically established near lava tube caves, leveraging their natural shielding from radiation, micrometeorite, and extreme temperature fluctuations [1]. A schematic of a lava-tube-supported base is shown in Fig. 1. The base could serve as a comprehensive platform for fundamental science, including materials science, fundamental physics, and life sciences under unique lunar conditions, long-term monitoring and investigation of the lunar surface environment, *in situ* high-precision assessment of key lunar resources, and lunar-based astronomical observations and research. A comprehensive geophysical array incorporating electric and magnetic field sensors, heat flow probes, seismometers, gravimeters, and radar systems could be deployed for multi-physical field observations of the Moon (Table S1). This enables a multi-layered investigation of the lunar interior: map vertical structures in the shallow crust to address (mega)regolith structure, magmatic scale, and crustal fractures; resolving lithospheric thickness and deep structures (e.g. deep intrusions) at middle layers; and probing the core–mantle boundary and core dynamics at great depths (Fig. S4). Synthesizing these cross-scale data will allow the construction of a refined model of the Moon’s interior, providing fundamental insights into its formation and long-term evolutionary history. Due to their natural protective properties, lava tubes are ideal sites for both short-term missions and long-term human habitation. Conceptually, a lava tube cave suitable for human exploration may be organized into three functional zones: an entrance zone, a research zone, and a habitation zone. The entrance zone would accommodate access stairways, communication systems, as well as air-tight sealing and pressurization facilities. The research zone would support scientific investigations, experimental activities, and *in situ* resource utilization. The habitation zone would be dedicated to crew living, which enables a sustained human presence beneath the lunar surface. Such a fully functional lava tube base would provide critical support not only for the long-term operation of crewed lunar exploration program, but also for the scientific exploration of the interior structure of the Moon, aligning with the Moon Insight concept.

**Figure 1.**
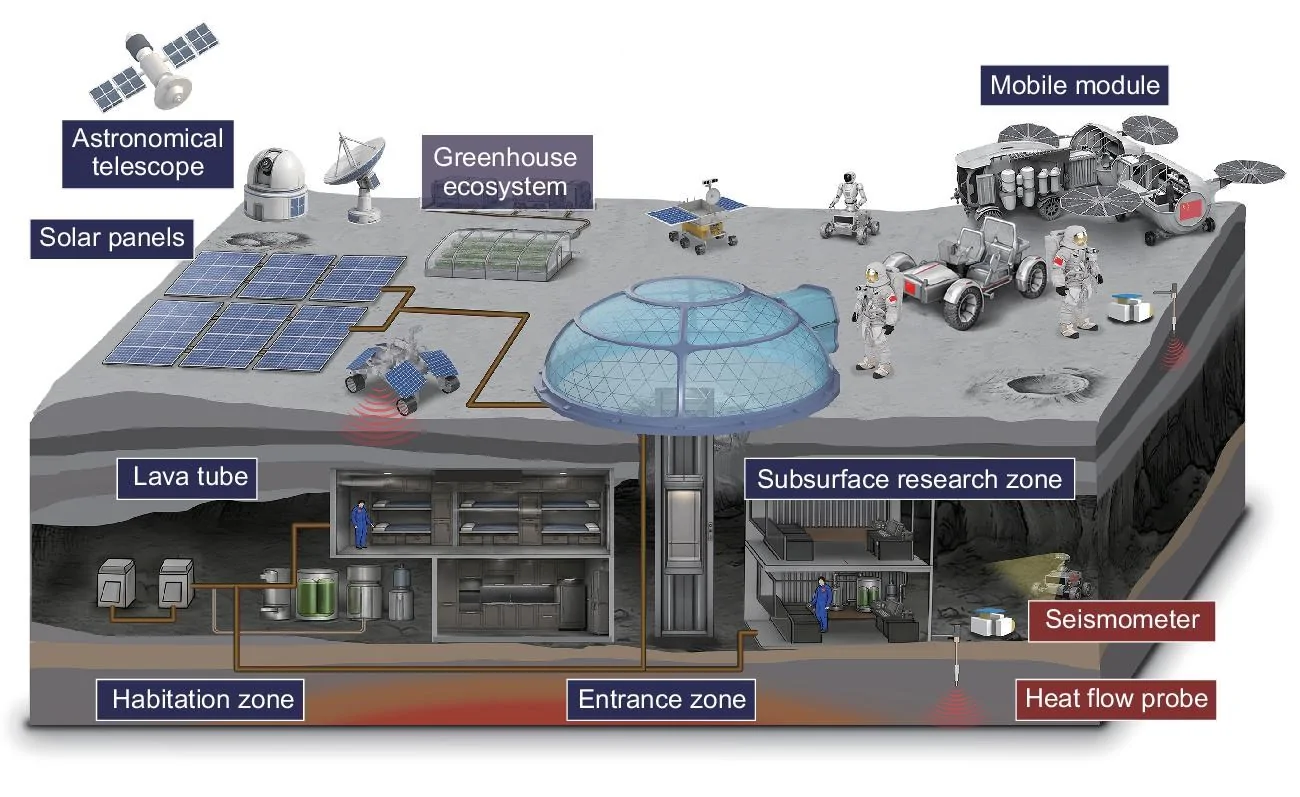
Schematic diagram of a lunar research station deployed based on a lava tube cave.

In summary, the ‘Moon Insight’ conceptual framework aims to probe the lunar interior and trace the history of lunar magmatic activity. Entering and exploring lava tubes represents a paradigm shift and a new milestone in lunar exploration, transitioning from surface observations to direct, *in-situ* investigation of the Moon’s interior. Also, the implementation of the ‘Moon Insight’ framework will provide broad opportunities for international collaboration by promoting shared scientific data, collaborative technology development, and joint scientific activities toward sustainable lunar exploration. By leveraging lava tubes as unique natural laboratories, the mission seeks to constrain the composition and stratification of the lunar mantle, characterize deep-seated magmatic processes, and reconstruct the Moon’s thermal and chemical evolution. Furthermore, lunar lava tubes are potential sites for future bases, providing ideal environments for both the scientific exploration and lunar resources utilization. Their enclosed, stable environments offer natural shielding and favorable conditions for long-term geophysical investigations, enabling high-quality measurements of seismic activity, heat flow, and subsurface structure. By combining geological observations with

sustained geophysical monitoring, lunar lava tubes have the potential to significantly advance our understanding of the Moon’s thermal and interior structures, while also offering promising opportunities for long-term scientific exploration research stations and in situ resource utilization.

## Supplementary Material

nwag474_Supplemental_File
