# Supplementary material for: Moon Insight: a conceptual framework for future lunar exploration
Source: Natl Sci Rev. 2026 Aug 5;13(16):nwag474. doi: 10.1093/nsr/nwag474 (PMC13532574; doi:10.1093/nsr/nwag474)
Supplement: Supplementary file 1 [file nwag474_supplemental_file.docx]

Supporting Material for

**Moon Insight: a Conceptual Framework for Future Lunar Exploration**

Yang Liu, Yongliao Zou*, Chi Wang*

National Space Science Center, Chinese Academy of Sciences, Beijing, China.

*Corresponding authors: Chi Wang and Yongliao Zou*

*[cw@nssc.ac.cn,](mailto:cw@nssc.ac.cn,) zouyongliao@nssc.ac.cn*

# Text S1. Introduction to lunar lave tube

Lava tubes are geological structures formed during volcanic eruptions, and their formation mechanisms have been documented in previous studies (Dragoni et al., 1995; Feng et al., 2024; Kempe & Ketz-Kempe, 2023; Sauro et al., 2020). As lava flows, its surface cools and solidifies, forming a mechanically sturdy and thermally insulating crust, while the underlying low-viscosity lava continues to flow. The initial lava tube can be further enlarged through thermal and mechanical erosion of the surrounding rocks by the hot flowing lava. Meanwhile, its insulating shell reduces hot loss, allowing the internal molten lava to be transported over great distance. After the magma source is exhausted, the left lava flow channel remains as hollow lave tube.

Due to limitations in observational techniques, lava tubes have not been directly observed on the Moon. Their presence is typically inferred from collapse-related landforms, such as pit (Figure S1), a negative landform characterized by near-vertical walls with inward-sloping rims (Robert V. Wagner & Robinson, 2014). Using the Terrain Camera images (~ 7m/pixel) of Kaguya mission, a pit feature was observed in the volcanic Marius Hill region, possibly representing a lunar lava tube skylight (Haruyama et al., 2009). Similar pit morphologies have since been widely identified across the lunar surface.

With the high-resolution images (0.5–2 m/pixel) collected by Lunar Reconnaissance Orbiter Camera (LROC) Narrow Angle Cameras (NAC), nearly 300 pit features have been cataloged on the Moon (<https://lroc.im-ldi.com/atlases/pits/list>) (R. V. Wagner & Robinson, 2021). Most of these are genetically relate to impact melt process, whereas only 16 pits are located in the mare region and 5 in the highlands (R. V. Wagner & Robinson, 2021, 2022) (Figure S2). These mare pits can reach widths of up to ~ 300 m and depths of up to 100 m. However, it should be noted that mare pit may also form through the collapse of voids associated with magma intrusion and tectonic activity (R. V. Wagner & Robinson, 2022).

In comparison to isolated pits, sinuous chain depressions in the mare region are generally considered more diagnostic indicators of collapsed lave tubes. An example is the ~48 km-long sinuous feature located near Gruithuisen K crater in the Procellarum basin (Figure S3).

Figure S1. Examples of lunar skylights formed due to collapse of lava tube ceiling at Mare Tranquillitatis (a) and Mare Ingenii (b). The white scale bar represents 100 meters. Mare Tranquillitatis skylight is 105 m in depth and 88–100 m in diameter. Mare Ingenii skylight is 80–88 m in depth and 130 m in diameter.

Figure S2. The 16 pit features identified in the mare region (R. V. Wagner & Robinson, 2021). The labels in white and yellow designate the name of lunar basins (Wilhelms et al., 1987) and pit features (R. V. Wagner & Robinson, 2021), respectively.

Figure S3. The spectacular sinuous landform is inferred to be a collapsed lava tube, located near Gruithuisen K crater in the Procellarum basin.

Figure S4. Schematic diagram of the internal structure of the Moon, highlighting poorly constrained crustal and mantle thicknesses, and unresolved parameters regarding the core's dimensions and state (solid/liquid).

Table S1. Instruments can be deployed for multi-physical field observation of lunar research station.

| Instrument | Exploration objectives | Effective investigation depth / Sensitivity range |
| --- | --- | --- |
| Gravimeter | Measure the gravity field and its temporal variations; invert subsurface density structure; identify local mass anomalies (e.g., magmatic intrusion); jointly constrain crust–mantle structure and tidal response with orbital gravity data | No fixed depth (integrated field response); primarily sensitive to ~1–50 km scale density variations under station-scale deployment |
| Magnetometer | Measure local magnetic fields and anomalies; investigate the origin and distribution of crustal remanent magnetization and magnetic minerals; probe deep interior structure via solar wind–induced fields | Primarily sensitive to ~1–10s km crustal magnetic sources |
| Ground Penetrating Radar (GPR) | Probe shallow dielectric structure; identify regolith thickness, stratigraphy, lava flow units, faults, voids, and shallow rock bodies | Several meters to up to 1000 meters |
| Magnetotelluric Sounder (MT) | Invert subsurface electrical conductivity structure; constrain thermal state, composition, and volatiles; reveal deep structure and potential partial melt zones; study electrical properties of the lunar core | Several to 200 km in typical; extendable to >1000 km depending on frequency band and observation duration |
| Seismometer | Record natural and artificial seismic sources; invert elastic structure; determine crustal thickness, mantle structure, low-velocity zones, and potential partial melt regions; characterize lunar seismicity and tectonics | Several to 100s km in typical; potentially sensitive to deep mantle and core (~1000 km scale) |
| Heat Flow Probe | Measure temperature gradient and heat flow; constrain internal heat flux and distribution of radioactive elements (U–Th–K); investigate lunar thermal evolution and its present-day thermal state | Direct measurement to up to ~10 m; indirectly constrains lithospheric thermal structure (~100 km scale) |

**References**

Brisset, J., Miletich, T., & Metzger, P. (2020). Thermal extraction of water ice from the lunar surface - A 3D numerical model. *Planetary and Space Science*, *193*, 105082. https://doi.org/10.1016/j.pss.2020.105082

Dragoni, M., Piombo, A., & Tallarico, A. (1995). A model for the formation of lava tubes by roofing over a channel. *Journal of Geophysical Research: Solid Earth*, *100*(B5), 8435–8447. https://doi.org/10.1029/94JB03263

Feng, Y., Pan, P.-Z., Tang, X., Wang, Z., Li, Y., & Hussain, A. (2024). A comprehensive review of lunar lava tube base construction and field research on a potential Earth test site. *International Journal of Mining Science and Technology*, *34*(9), 1201–1216. https://doi.org/10.1016/j.ijmst.2024.06.003

Haruyama, J., Hioki, K., Shirao, M., Morota, T., Hiesinger, H., van der Bogert, C. H., et al. (2009). Possible lunar lava tube skylight observed by SELENE cameras. *Geophysical Research Letters*, *36*(21). https://doi.org/10.1029/2009GL040635

Kempe, S., & Ketz-Kempe, C. (2023). Pyroducts, Genesis and Internal Development and Their Possible Link to Lunar Rills (Vol. 2697, p. 1006). Presented at the 4th International Planetary Caves Conference. Retrieved from https://ui.adsabs.harvard.edu/abs/2023LPICo2697.1006K

Liu, J., Zhang, S., Ouyang, Z., Zhu, K., & Zeng, X. (2025). The key scientific and technological issues in lunar resource exploration. *CHINA MINING MAGAZINE*, *34*(1), 1–8. https://doi.org/10.12075/j.issn.1004-4051.20250126

Liu, J. Z., Li, X., Zhu, K., Xu, Y., Yang, Y., Zhang, J., et al. (2022). Key science and technology issues of lunar in situ resource utilization. *Bulletin of National Natural Science Foundation of China*, *36*(6), 907–918. https://doi.org/10.16262/j.cnki.1000-8217.2022.06.005

Pei Z., Liu J., Wang Q., Kang Y., Zou Y., Zhang H., et al. (2020). Overview of lunar exploration and International Lunar Research Station. *Chinese Science Bulletin*, *65*(24), 2577–2586. https://doi.org/10.1360/TB-2020-0582

Rayman, M. D., & Saunders, R. S. (1996). Optical interferometry from the lunar surface. *Advances in Space Research*, *18*(11), 45–48. https://doi.org/10.1016/0273-1177(96)00087-7

Sauro, F., Pozzobon, R., Massironi, M., De Berardinis, P., Santagata, T., & De Waele, J. (2020). Lava tubes on Earth, Moon and Mars: A review on their size and morphology revealed by comparative planetology. *Earth-Science Reviews*. https://doi.org/10.1016/j.earscirev.2020.103288

Shaw, M., Humbert, M., Brooks, G., Rhamdhani, A., Duffy, A., & Pownceby, M. (2022). Mineral Processing and Metal Extraction on the Lunar Surface - Challenges and Opportunities. *Mineral Processing and Extractive Metallurgy Review*, *43*(7), 865–891. https://doi.org/10.1080/08827508.2021.1969390

Wagner, R. V., & Robinson, M. S. (2021). Occurrence and Origin of Lunar Pits: Observations from a New Catalog (p. 2530). Presented at the 52nd Lunar and Planetary Science Conference. Retrieved from https://ui.adsabs.harvard.edu/abs/2021LPI....52.2530W

Wagner, R. V., & Robinson, M. S. (2022). Lunar Pit Morphology: Implications for Exploration. *Journal of Geophysical Research: Planets*, *127*(8), e2022JE007328. https://doi.org/10.1029/2022JE007328

Wagner, Robert V., & Robinson, M. S. (2014). Distribution, formation mechanisms, and significance of lunar pits. *Icarus*, *237*, 52–60. https://doi.org/10.1016/j.icarus.2014.04.002

Wilhelms, D. E., McCauley, J. F., & Trask, N. J. (1987). *The Geologic History of the Moon*. Washington DC: U.S. Government Printing Office.
